# The distinctiveness of human aging II: social processes and the regulation of aging

**DOI:** 10.1007/s00391-026-02609-7

**Published:** 2026-08-12

**Authors:** Dale Dannefer, Jessica Kelley, Olugbenga Okunade

**Affiliations:** Department of Sociology, Case Western Reserve University, 10900 Euclid Avenue, Cleveland, OH USA

**Keywords:** Labelling, Activity theory, Organismic paradigm, Ageism, Nursing homes, Labelling, Aktivitätstheorie, Organismisches Paradigma, Ageism, Pflegeinrichtungen

## Abstract

This paper builds upon the principles and dimensions presented in the paper, “The distinctiveness of human aging I”, which is intended  to provide a foundation for apprehending and understanding variation in individual and population patterns of human aging. Given the clearly established role of contextual factors and context-related dynamics as key explanatory factors to account for such variation, this paper sets forth  key dimensions of the socioenvironmental regulation of processes of human aging and their interactions across micro, meso and macro levels of social organization. We focus on one specific domain of age-related challenges and one (albeit broad) institutional domain: rapid population aging and on the institutional level, the attendant expansion of geriatric diagnoses and elder care institutions, focusing especially  on nursing homes. Through this discussion, we demonstrate that human aging entails a distinctly flexible and in key ways modifiable set of processes. It is thus continually reconstituted for individuals and for social groups and populations as existing social arrangements are continuously reproduced, regardless of the value (positive or negative) of such arrangements for human aging.

## Abstract (de)

Dieses Papier baut auf dem vorangegangenen Beitrag „Zum distinktiven Charakter menschlichen Altern I: Anthropologische und entwicklungsbezogene Grundlagen“ auf, in dem die Grundlagen des Begreifens und Verstehens von Variationen individuellen und populationsbezogenen menschlichen Alterns erörtert wurden. Ausgehend von der klar etablierten Rolle von kontextuellen Faktoren und kontextuellen Dynamiken zur Erklärung von Variationen menschlichen Alterns werden im vorliegenden Beitrag zentrale Dimensionen der sozioumweltbezogenen Regulation von Alternsprozessen im Humanbereich sowie interaktive Prozesse entlang von Mikro‑, Meso- und Makroebenen sozialer Organisation behandelt. Wir fokussieren uns auf einen bestimmten Bereich altersbedingter Herausforderungen und einen (wenn auch weit gefassten) institutionellen Bereich: die rasche Alterung der Bevölkerung und den damit einhergehenden steigenden Bedarf an Pflegepersonal sowie, auf institutioneller Ebene, die damit verbundene Zunahme geriatrischer Diagnosen und von Altenpflegeeinrichtungen, wobei wir uns hier auf Pflegeheime konzentrieren. Dabei zeigen wir, dass humanes Altern durch spezifische Formen von Flexibilität und Veränderung gekennzeichnet ist. Altern kann damit als kontinuierliche Rekonstitution auf der individuellen, sozialen und populationsbezogenen Ebene verstanden werden—unabhängig davon, ob diese sich reproduzierenden Arrangements mit eher positiven oder negativen Aspekten menschlichen Alterns verbunden sind.

This paper develops further the examination of aging began in the prior paper, “The distinctiveness of human aging I: anthropological and developmental foundations”. In that paper, it was demonstrated that the developmental and aging processes of humans manifest some distinct features and dimensions compared to the aging of other life forms. In particular, humans are constitutionally “world-open”—hard-wired for flexibility*—*in ways that set them clearly apart from other species, and even from other primates, from birth onwards [7, 58, 61]. The resultant diversity of human skill sets and human societies prompted Robert Sapolsky to observe, as we further noted, that “… the nature of our nature is not to be particularly constrained by our nature” [63].

Yet if the nature of human nature is not rigidly structured by ontogenetic processes or otherwise predetermined behavior patterns or developmental stages, how is it that virtually every human society manifests a clear patterning and orderliness with respect to multiple domains of experience, including age? From the abundance of evidence provided  in the earlier paper, it is clear 1) that much of the observed variation in patterns of individual human aging both within and between societies is attributable to differences in individual experience and 2) that differences in experience are not random, but are socially and culturally organized, and thus vary in predictable ways across social contexts. Indeed, the orderliness in patterns of aging characterizing a given social setting contrasts with the great variation observable between social contexts. It is also clear that human action contributes to aging and changes in patterns of aging in ways that are quite unique to our species.

In any given social context, such factors operate largely unnoticed throughout the life course, from their internalization in earliest childhood to advanced old age. Although human actors who are engaged in daily routines and struggles typically give little attention or thought to the social forces that organize their lives, such forces are relentless and provide the structures that organize such routines. The continuous operation of social forces can be analytically discerned at multiple distinct system levels. They are part of “the waters we swim” [26] and are embodied and internalized in what Bourdieu [10] terms *habitus*, the context-specific set of dispositional practices that are internalized by members of a social order, including practices that organize the experience of aging, beginning early in the life course. Thus, in rural Guatemala it is “normal” and taken for granted to assign the task of infant care to 5‑year-old siblings and in other indigenous societies  to train even younger children  to use fire or machetes [45, 61], practices that would have modern parents arrested for child abuse or maltreatment.

Whether deemed healthy or unhealthy, the effects of such practices pervade physiological and psychosocial aspects of development and aging. Thus, a key to understanding aging is to begin to apprehend the scope of the relevant elements and dimensions of context.

While space does not permit anything like a full treatment of such factors, we present here a discussion of some key dimensions of the socioenvironmental regulation of processes of human aging. Such processes can readily be seen to operate at multiple levels of social systems, from the most basic and universal micro-level of face-to-face interaction to the elaboration of macro-level factors of the place of age in the organization of large-scale societies and indeed international relations.

## The constitution of human aging in micro-interaction

The social organization of human experience begins at the micro-level, a fact that is self-evident, as many relatively small-scale indigenous societies have long existed and some still endure without a macro-level, but nevertheless with a manifest order that has distinct and intricate forms of social organization, including the organization of development and aging. It is also self-evident for individuals: parental and social nurture are essential to enable the neonate to survive and engage in society.

In modern nation states where age is often used as a principle of social organization and structures daily lives in ways that are imposed from above, the experience of aging is still constituted or realized in ongoing social processes at the micro-level. Even if what is deemed appropriate or possible is in many respects defined by the state (e.g., being in school when one is 9 years old, or confronting mandatory retirement at an arbitrary age), it is enacted and brought to reality at the micro-level, as actual persons interact with each other, all the while reinforcing or occasionally challenging societal expectations of what is “normal” given one’s age. Such processes operate continuously, in virtually every domain of society, from media production and advertising to medical diagnostics and nursing care, to the economics and norms of everyday lifestyle options. Through such interactions, humans receive a constant stream of implicit messages about how their particular age situates them, whether through displays of deference to elders or through the conduct of store clerks who are dismissive or impatient with older shoppers. Such daily experiences are involved in the very constitution of human aging. Extreme deference to older persons in one society reflects an internalized cultural value that is integral to its habitus, just as does the dismissiveness of the store clerk in a different society. Neither posture is inherent to the aging organism; both are socially organized.

To illustrate our points regarding the interactive micro, meso and macro processes that serve to reinforce the social organization of age in a given society, we focus on one specific arena of age-related challenges and one (albeit broad) institutional domain: rapid population aging and the concomitant growing demand for elder care work, and on the institutional level, the attendant expansion of geriatric diagnoses and elder care institutions, focusing here on nursing homes.

**Fig. 1:**
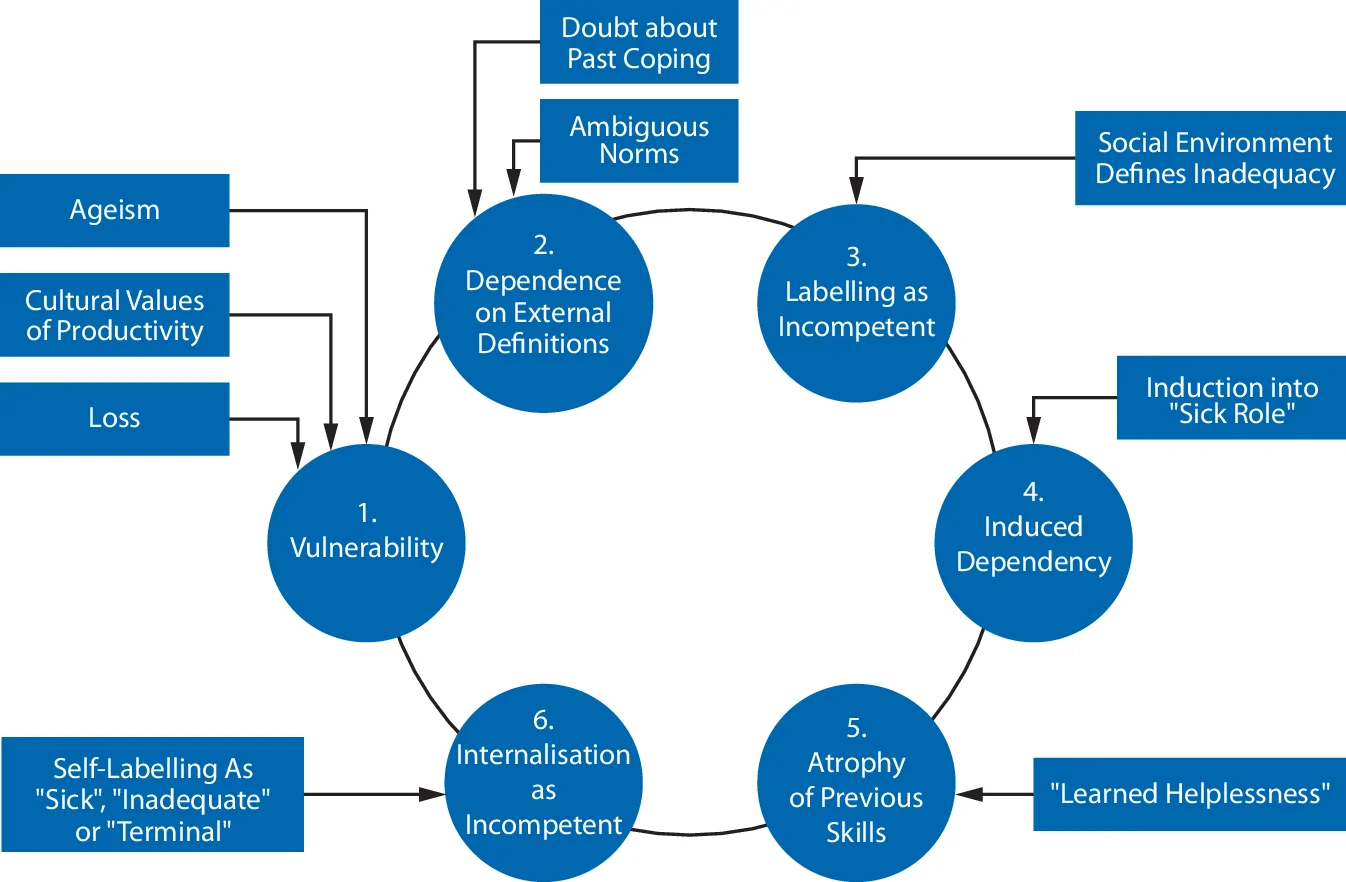
Bengtson’s [6] cycle of induced incompetence. Also, Falletta and Dannefer [36]

A solid starting point for considering some of the micro-level dynamics that shape the nature and experience of age is offered in Vern Bengtson’s classic “cycle of induced incompetence”, shown in Figure 1 [6]). The “cycle” illustrates how the dynamics of interaction in everyday conversation inevitably involve appraisal of others. When dealing with older people in contemporary Western societies, this very often includes the mobilization of ageist biases and presumptions, and feelings ranging from ambivalence and hostility. Thus, as shown in Fig. 1, we begin with the circumstance of living in society with views of older adults that include assumptions of incompetence and decline. Such assumptions are part of habitus, and are readily mobilized by others and used broadly to interpret the conduct of older persons, whether neighbors, relatives, customers or patients. This is not exclusively a modern phenomenon, nor a process fully external to the older adult actor. Indeed, eighteenth-century writer Samuel Johnson’s reported observation demonstrates the prevalence of such views, warranted or not, and their internalization even by elders themselves.

“If a young man mislays his hat he says he has mislaid his hat. The old man says,

*‘I have lost my hat. I must be getting old.’” *(Kushner 52)

Now much more than three centuries ago, such age-specific inferences often seem like a plausible account to everyone involved, including older persons. Even if such assumptions are without actual physiological foundation in a given individual case or have a spurious cause, the appraisal of others and the power of the messages of the habitus can be difficult to reject, an example of what Bourdieu and Burawoy  call *symbolic violence* [12, 16].

This process of internalizing the possibly unfounded views of oneself held by other people—a key component of what is known as *labeling* in sociological research and theory—entails dynamics that are subtly present in much social interaction. As has been demonstrated in other contexts (including school, work and family dynamics), Bengtson’s cycle illustrates how the internalization of dependency can be a matter of self-fulfilling prophecy in medical or elder care settings, when family members or medical professionals selectively interpret incidental or benign behavior as “the beginning of the end” and, given the power and presumed expertise of medical professionals, that message may be internalized by the older person in question, discouraging their own exercise of their competence and potentials.

In the case of individuals living in long-term care institutions, strong empirical evidence of the power of such labeling dynamics in everyday interaction has been provided by Margaret Baltes and Hans-Werner Wahl’s [4] ingenious study of “dependency-support scripts”, via which caregivers and others often incongruously and gratuitously treat older persons as in need of care and support, whether or not such a need actually exists. If an individual does not have such needs, they may be created in them by the imposed assumptions and treatment of staff. Baltes and Wahl’s rigorous analysis provides a strong quantitative basis that supports other, more descriptive accounts of how dependency is encouraged in such settings (see, e.g., Dannefer, [28, chapters 9–10], 33, 42, 50, 68).

## The constitution of human aging in meso-level processes

Staying with the domain of long-term care, the impact of meso-level social system forces can be illustrated by considering the impacts of the structural organization of nursing home life, and the attendant culture. In the US and elsewhere, nursing homes provide the default provision of care for frail and cognitively impaired elders.

For the better part of a century now, the standard template of nursing home life in modern societies (and perhaps especially in the US) entails a regime of medical and nursing expertise imposed on the residents, typically in large, hospital-like warehouses (although called nursing *homes*). In such settings, residents’ daily existence is defined in terms of need and compliance with medical and behavioral routines. It thus operates as a total institution, which is the bounded context within which the lives of residents are confined, roles are tightly scripted, and powerful others control the organization of residents’ daily experience [41, 68].

Thus, the physical layout and the imposed schedules, policies and rules of the nursing home provide a meso-level framing that sets the parameters of life in the nursing home context for residents, staff and visitors as well. Residents are structurally positioned and formally defined as existing only as passive recipients of care, as individual actors upon whom no demands are made. It is a context within which the Baltes and Wahl’s dependency-support script is a typical form of communication, as knowledgeable experts and professionals provide “services” to residents, who are expected to do nothing and to know nothing but to comply with the “expert” knowledge imposed on them.

Available evidence demonstrates how the daily routines of nursing home life both reflect and reinforce dependency and powerlessness. In conventional US nursing homes, for example, it is common practice for meals to be served in a large dining room on a precise time schedule, and where each resident has a preassigned seat, at a table with the same seatmates for every meal. Similarly, bedtime as well as getting up are largely determined by an imposed schedule over which residents have no control.

Such practices are part of the standard nursing home culture, informing the entrainment and “work socialization” of staff, and reinforcing a view of residents as passive and without opinions or what is often called agency. Moreover, even in the face of evidence indicating that nursing home residents have more potentials and capabilities than they are given credit for, the temporal pace and constraints of institutional processes do not encourage or allow  for the realization of those potentials. A recent report from a study of an excellent US nursing home includes this account from a participant-observer researcher, the late Janet Gelein, who was also drawing on her skills as a licensed nurse practitioner:

“… Picking up Emily’s spoon I filled it … As I moved the spoon close to her mouth she opened it and accepted the food … After she had finished several mouthfuls, I initiated a conversation with other people. As we talked I … (included) Emily in the conversation …. as the meal progressed I started to wonder if Emily really did need to be fed. ‘You seem interested in this food, Emily’, I said. ‘I wonder if you can feed yourself?’ She did not respond, but as I enclosed her hand within mine to use the spoon, Emily did not resist. After doing this several times, together, I complimented her efforts. Noticing that she seemed to like the pudding best, I moved it directly in front of her, saying: ‘Why don’t you … eat that on your own’. Reluctantly at first, but with increasing confidence, she continued feeding herself the entire dish of pudding …” [28].

If this is a telling episode, it is at least equally telling to consider the reaction of work staff, and also the facility administration when this episode was reported to them. The response was resoundingly, “yes, we know, but we don’t have time.” As demonstrated through the ethnographic work of Diamond [33] in nursing homes, the organization of staff time is tightly controlled, over-scheduled, and focused on efficiency in the delivery of care. With dozens of residents to feed and care for in a narrow window of institutionally defined clock time, facility staff recognized such potentials but reported feeling constrained by the imposed structure of institutional routines and survey (evaluation) criteria.

Staff experiences of stress and overload seem to be part of the standard expectation in such contexts. In the USA, a majority of nursing homes are chronically understaffed, failing to meet federal staffing standards (e.g., [67]). Thus, staff reactions are understandable as part of an institutional regimen that is beyond staff and in some respects beyond administrative control. Less recognized is the contribution of such shortages to a general culture of ageism and perhaps even more, there is little recognition that part of what is occurring in such settings and in episodes like the above encounter of Janet and Emily is the reproduction and reinforcement of dependent behavior and the concomitant reinforcement of negative subjective self-appraisals.

Of course, life in such a medicalized setting represents a pervasively taken-for-granted situation or even “life stage” in Western late modern societies. Given its pervasiveness, it is often little questioned. It is thus understandable that the industry has been slow to recognize that this very organizational form and the accompanying culture often has adverse effects upon the capabilities of the residents who are subjected to it, often involuntarily. It further exemplifies the broader cultural forces that shape how older adults are viewed (in many Western societies) as older adults approach the end of life, such that dependency and incompetence become a form of master status.

Yet the impact on residents is multidimensional. Living in a nursing home has been empirically demonstrated to aggravate dependency and shorten lives [14, 21, 35, 44]. To the extent that dependency and incompetence are exacerbated or even generated by such social arrangements, their attribution to organismic aging represents a form of *naturalization*, misattributing socially produced phenomena to biological nature.

An especially telling demonstration of the power of social organization to alter the life trajectories of elder residents by the industrially modeled regime of conventional nursing home life has been provided by an examination of what happens to resident health and longevity when the institutional context is reorganized in more humanly responsive and “resident-directed” terms. Changing the physical layout and organizational processes to allow for a more “homelike” atmosphere and routine was followed by a drop in adverse health events [30]. Such findings are consistent with and build upon the classic work of Langer and Rodin [53] which demonstrated the health benefits of giving nursing home residents more discretion, responsibility and menaingfulness in daily activities.

A significant example of such innovation comes from a study of an ambitious effort at culture change in a US long-term care facility. Changes were implemented that allowed residents to have control over such matters as when to get up in the morning, when to have breakfast and what to eat (with an option to make their own breakfast), and more control over social interaction at mealtimes. Other resident-friendly changes included “aging in place”, enabling residents to remain in the same room and same unit rather than progressing through the “resident career”, typically requiring relocation to a skilled nursing or memory care unit when a person’s needs changed. Such organizational innovations were followed by dramatic reductions in mortality rates and reductions in common health issues such as infections and falls [30]. Such changes have also been seen in other settings, along with reductions in medication use [13, 56, 66, 70].

Such welcome changes, reversing or slowing trajectories of decline, are entirely consistent with self-determination theory [32] which posits three basic human needs (competence, autonomy and relatedness) all of which are dramatically violated by the conditions of everyday living imposed upon many nursing home residents. It is noteworthy that these three posited needs correspond closely to what leading American nursing home reformer Bill Thomas identified as the “three plagues” of nursing home life, helplessness, boredom and loneliness [66, 70]. Loneliness obviously is a problem of relatedness, helplessness is a typical experience when there is a lack of opportunities to demonstrate competence, and boredom when one has little control or autonomy over one’s option for activities.

Dealing with such contradictory and paradoxical realities inevitably direct attention to forces beyond the local nursing home, to the broader cultural, political and economic  developments that shaped its form as an institution.

## The constitution of human aging in macro-level processes

No known evidence suggests that the vibrancy of the three basic human needs of competence, autonomy and relatedness diminishes with age. To assume otherwise would be a presumptuous and destructive form of ageism, and clearly one without warrant. Yet precisely such a mindset is reflected in the social, political and economic assumptions that led to the widespread implementation of the modern nursing home in the US, informed and greatly expanded by the Older Americans Act of 1965 and related initiatives.

It was in reaction to the broader zeitgeist of old-age discrimination reflected in such large-scale initiatives that Robert Butler coined the term “ageism” (in a 1968 *Washington Post *interview with Carl Bernstein [17]). His concerns were elaborated in *Why survive: the tragedy of being old in America, *Butler’s Pulitzer Prize-winning critique of ageism as pervasive cultural and institutionalized force, published in 1975 [1, 8, 57]. As many readers will know, *Why survive *forcibly demonstrated the pervasiveness of the unwarranted assumption that individuals’ needs are reduced when they reach “old age”, and its mismatch with the realities of aging.

Ageism is powerfully reflected in the presumptions of policymakers, medical experts and politicians that older people would be well-served or would view positively the prospect of being warehoused in a rigidly institutionalized environment, one that is typically  temporally and spatially constrictive and barren, socially as well as physically. As one cannot presume that such decision-makers were actively trying to bring harm to those that the modern nursing home is designed to serve, it seems that the remaining logical explanation, consistent with Butler’s initial 1975 analysis, is an unreflected and habituated system of assumptions that older people need only to be taken care of. This broad assumption seems indeed to have become part of the modern habitus [18].

Such an assumption, of course, renders invisible the need to consider the dynamic and social nature of the basic human needs posited by self-determination theory, thus implicitly assuming such needs somehow evaporate with age.  Despite its lack of any evidentiary support, this assumption remains pervasive in contemporary discourses about older people, including discourses that shape the mindset of policymakers and that inform policy. The humanly destructive inability to nurture residents’ potentials described in the prior, meso-level section is to a considerable extent a consequence of policies that reflect this cultural mindset.

These “dependency debates”, now occurring worldwide, provide fresh evidence that growing proportions of older adults in a society are defined as a “problem” that needs to be solved, often through a series of significant structural or economic changes such as relaxing immigration restrictions to increase availability of younger workers, taking on added national debt to finance public pensions, raising retirement ages or privatizing risk in old age (see Goerres and Vanhuysse [40] for a comprehensive global review).

The above examples, grounded in largely Western contexts where older adults are subject to cultural devaluation, can be counterposed with examples from other parts of the world where old age is still considered to be valued status, especially within one’s family. In the case of South Korea, grounded strongly in filial piety, the government built a long-term care insurance program that centers on supporting families who are caring for their older relatives by providing home health care services, caregiver assistance, and robust community-based alternatives to institutional nursing homes [37]. While these efforts are not without tension, framing the social need as supporting families who expect to care for their aging relatives is a cultural value that then translates into the macro policy structure of long-term care [73].

While there is no question that labor force, pension systems, housing, and long-term care all pose challenges for countries with populations that are rapidly aging, it is also clear that an implicit ageism shapes debates on these issues, deflecting a recognition of the actual physical and cognitive potentials of aging individuals. At the macro-level, pervasive cultural assumptions about aging and older adults continue to be reinforced as part of both everyday popular discourse and high-level policymaking [43]. These ageist assumptions are not only a vibrant part of contemporary popular culture but are often central to medicine including geriatrics, and indeed, even gerontology [28especially chapters 1 and 9]. That said, myriad examples abound to demonstrate that different cultural assumptions about aging across societies can result in widely varying micro, meso and macro processes within societies.

## The distinctive character of human aging: implications for theory and research

In this final section, we consider what the distinct features of *homo sapiens* imply for advancing a general and theoretically grounded understanding of human age and aging. This discussion encompasses both the basic principles and considerations set forth both in the the prior paper (part 1 of this 2-article statement), and the social and historical dynamics that are both generated by those basic features, and act back upon them. It seems clear that any theoretical formulation that seeks be serious about “respecting the reality of its subject matter”, as science is obligated to do [9], cannot ignore these structural features of human aging.

A logical preliminary step in developing a comprehensive understanding of human aging is to clarify those aspects of aging that recur independent of sociohistorical contexts, and those that are contingent and subject to experiential and contextual influence. Some key features of development and aging, such as the development of secondary sex characteristics are indeed universal (even though timing may shift). But the development and progression of movement patterns and skills in early childhood are highly specific to the habitus of a social setting, whether in the development of abstract math skills or learning to hunt forest game, As the analysis moves into adulthood and later life, the patterning of age-related characteristics manifests wide diversity, reflecting the distinctively human conditions of inherent flexibility and interactivity with context. Comparative research designs across societies and between cohorts over time in the same society demonstrate high degree of variability in the patterning of individual aging, both physically and psychologically, and from early childhood onward. As reviewed earlier, some societies expect children of 5 or 7 years old to engage in productive work involving high responsibility or danger; other societies impose retirement and have even expected “disengagement” at an arbitrary age like 65 years. Thus, comparative evidence compels us to recognize that organismic, deterministic models of aging are unrealistically rigid and oversimplified. Such comparative evidence needs rigorous design grounded in the theoretical underpinnings of the constitutive nature of social worlds with the ability to compare humans in different contexts. We elaborate on this point in the remaining part of this section.

Broadly, it is intriguing to consider that despite dramatic variation, the prevailing cultural system of virtually every society manifests in its institutions and practices a particular understanding of age or stage-related values, even if age is not calibrated in years or other quantitatively precise units of measurement. Thus, whatever form it takes, it will be a form that has significant implications for human development and aging, providing and/or limiting opportunities for continued growth, development, and achieving potentials. Such culture-specific understanding can be detected at a population or policy level, but remaining at the macro-structural domain may obscure the realities of “life on the ground” [27], where individuals encounter the stratified opportunities and constraints that ultimately produce a diverse range of outcomes within societies even while simultaneously producing new population-level trends.

So where does this leave us with respect to advancing an understanding of age and aging? We quickly review the interacting types of forces we have identified.

First, it is clear that the role of ontogenetic, preprogrammed sequences of developmental change, while ubiquitously present and powerfu in accounting for certain types of age-related change, are nevertheless relatively limited in directing behavior and conduct compared to what hass often been assumed to be the case, and in comparison to what is found in  many other species. Instead, human ‘hard-wiring for flexibility’ [61] and the resultant “world-openness” [7] comprise a foundation for multiple potentials and possible directions of human development, and patterns of aging. This sociosomatic condition can readily be seen in the wide range of patterned variation in human growth and aging, and health and longevity that are evident across the planet. As we noted earlier, such person-context interactions acquire a new order of magnitude with the discovery of epigenetics and related processes involving the environmental conditioning of gene expression.

Second, it is abundantly clear that lacking such organismically programmed patterns, human growth, change and aging processes do not merely follow a random or purely volitional direction, but are deeply structured from prenatality onward by culturally entrenched and imposed patterns of local interaction that are internalized as dispositions, what Bourdieu [11] calls *habitus*. This general process entails first the internalization of language and embodied cultural practices, which from the beginning entail the learning of a culture-specific take on the empirical and social world. The social world, in turn, consists of multiple levels of social organization that interact to sustain and reproduce these patterns, as integral and systemic features of any viable context and culture.

Third, humans are perhaps most distinctive in the exercise of intentionality, purposefulness, and generative social action, which always carries the potential for creativity [26, 29, 48). These are essential elements in the process of constituting the social world itself. Throughout human history and in every culture, generative action has been integral to constituting the social dynamics reviewed above and to the constitution of social worlds, which includes, of course, the manufacture of culture-specific patterns of aging. Most such human activity and interaction is undertaken with no thought to its role in constituting aging; however, one notably distinctive line of action undertaken by humans is the pursuit of deliberate efforts to exert control over our own aging, which has many manifestations (e.g., anti-aging dietary experiments and claims, age-friendly policies).

As essential features of human aging, these three conditions (ontogeny, habituation and generative action) built upon the foundation of the “world-open” nature of the human organism (as described in the first paper) thus provide a minimal framework for apprehending human aging. It should quickly be evident that by these criteria, many dominant approaches to theorizing aging across the sciences cannot do justice to the empirical reality of human aging.

While space does not permit a comprehensive review of extant theoretical approaches to aging, we offer here a few observations on the implications of this analysis for some popular approaches to conceptualizing aging. As we begin to undertake this task, a first thing to note is that what are generally seen as the dominant and robustly popular theoretical approaches in gerontology at best, satisfy only some of these criteria, and often do so relatively weakly. These paradigms are frequently implicit, rather than explicit, in the design of such studies. Inherent assumptions of socio-emotional selectivity and withdrawal from society as we age, for example, may drive expectations of potentials among older adults under study.

Let us illustrate this point by tracing the development and application of theories of aging in gerontological research. The classical organismic paradigm*,* which assumes internally driven, sequentially ordered and universally determinate life stages, has long been positioned as a dominant theory of aging and the life course, well represented in medical approaches to aging and in social gerontology by Cumming and Henry’s disengagement theory, and in midlife by the propositions of various sequential theories of adulthood (e.g., [55]), following structural theories of early life as articulated by, for example, Piaget [60], Killen and Smetana [51] in early life. While such organismic assumptions obviously are exactly correct for many species of life, they do not recognize the importance of socially organized habituation, i.e., of social context, and also of human generativity (creativity and intentional action, agency) [48] which acts back upon the person and upon processes of aging.

A number of critiques of the organismic paradigm as applied to human development and aging have been offered, [15, 19, 24, 28, 39, 46, 59, 65]. While the validity of such critiques has been acknowledged, the organismic approach remains popular and highly influential, albeit often in implicit form, such as in “normative” age-graded assumptions manifest in fields like education and medical practice.

The life-span perspective effectively challenged the adequacy of the organismic perspective by confronting the ontogenetic paradigm head-on, with evidence of important cohort differences in how individuals develop and age (e.g., [3, 64]). This was a crucially important step in opening the door to a recognition of context via history and cohort but it was also limited, as beyond the cohort-historical axis, it lacked the conceptual tools to organize an understanding of context except via a “normative/nonnormative” dichotomy, thereby privileging an ill-defined “normal” or “normative” state, and essentially treating synchronic contextual variation as random or even deviant (see, e.g., [24, 28, 59]).

The much celebrated activity theory approach in social gerontology focused the explanation on the beneficial value of purposeful activity in maintaining a healthy approach to aging [34, 49]. While activity is centrally important, the term “activity theory” as generally applied in gerontology also targets only one of the dimensions of aging. It countenances neither the importance of ontogeny nor the reliance of activity itself on socially organized meaning structures. Even more, it fails to recognize that human action does more than express the needs and perhaps provide some sense of agency and self-assertion to the actor. Typically without the actor’s intention or awareness, human action creates and reconstitutes relationships and patterned activities in the social world and thus participates in reconstituting that very world. It thus is more potent and consequential that the focus on individuals and individual action alone typically recognizes.

Thus, the self-society relation cannot be adequately grasped simply by thinking in terms of contextual effects on individuals or even by adding to that the idea that individual action influences others, or produces one’s own development [54]. Because it engages and participates in the ongoing constitution and reproduction of social worlds, an adequate grasp of the significance of human action requires that it be recognized as constitutive of all social life. Thus, it has within it the potential and force to shape and reshape social relations and arrangements, and sometimes does, often unintentionally, but in some cases intentionally as well. Thus, the popular view of individual “agency” as “composing a life” [5] or “producing” one’s own development, while not false, significantly underestimate the efficacy potentials of human development and aging.

This recognition invites a special or perhaps renewed attention to the character of social action and its relation to the social world, especially as its arrangements are relevant to aging individuals. Questions readily arise concerning an array of practical social issues that are impacting and will impact human aging, and that are under human control. Of course, climate change and its implications for future generations and current young people as they age comes immediately to mind, but so do an array of other issues. What are the implications of the finding that dementia rates are declining with continued growth in educational upgrading? Of artificial intelligence (AI) on the structure of major life-course institutions such as those that have organized schooling and careers, and taxation and transfer payments? Of course, a major concern of the developed world over recent decades has to do with the increased concentration of wealth, which is a social issue whose interaction with age is receiving growing attention, as the amplificatoin of the stratification of longevity becomes increasingly difficult to ignore [2, 20].

Over recent decades, it has become increasingly clear that the stratified character of many postindustrial and late modern societies intersects with age and the life-course in systemic, predictable ways. The general obdurate socioeconomic realities of entrenched stratification inherently entail amplification effects that lead to increasing inequality over the life-course, effects aggravated by neoliberal policies [23, 31]. The recognition of this enduing and robust reality has given rise to a focus on cumulative disadvantage/advantage processes, about which George and Ferraro wrote: “If there has been a bona fide theory based on the life course perspective, specifically the principle of life span development, it is cumulative advantage/disadvantage theory” [38, p. 5].

What cumulative disadvantage/advantage (CDA) adds to the above considerations is the recognition, central to a full understanding of human aging, that aging happens not just within people but between them [25, 28, 47, 62], because aging occurs as part of the reproduction of systems of social inequality. Inequality is socially organized not just at a given point in time, but also in the systemic production and allocation of stratified trajectories of aging individuals located at different points in social structure. The recognition that aging is interlinked with processes of social stratification opens up a new set of theoretical insights and research problems, yet by itself it does not elaborate the connections of the socially organized and stratified processes of aging to other age-related processes, such as those in the domains of ontogeny or of generative human action.

Although limited in scope as used in social gerontology, the term “activity theory” has a more broad and basic fundamental usage in efforts to apprehend human processes in the paradigmatic approach to learning and development introduced by Lev Vygotsky and well-articulated by those who are elaborating his approach. Recently, the potentials of the Vygotskian framework have been well illustrated In the work of Anna Stetsenko and associates in her “transformative approach” to human development and indeed, human possibilities [69]. This approach differs even from other critical and socially attuned frameworks because the focus is not simply on exposing and detailing patterns of social reproduction nor on analyzing individual adjustment to or cooptation by existing conditions. Rather, it is about deliberate and creative intentional action, but not action simply at the individual level of, e.g., adopting new exercise techniques or dietary interventions.   It thus envisions change-oriented, goal-directed action and interaction with the goal and possibility of providing positive transformation of the social arrangements and framing within which aging is experienced. It is an approach with some demonstrated success in dealing with developmental issues, especially in the earlier life course [71]. This returns us to primacy of the micro-level, and the issue of the active process of world-construction as an integral component of human aging, entailing both action and the construction of the systems within which patterns of aging are realized.

This transformative approach interrogates not only one’s personal lifestyle, but also the assumptions undergirding the collective practices of a society and the institutions that organize lifestyles, including both their humanizing and their destructive tendencies and potentials. The latter are evident in the growing body of work on aging and poverty, and on the adverse effects various fforms and manifestations of cumulative dis/advantage (e.g., weathering, abbreviated life course patterns) and contrasted at the other end of the social stratification spectrum by the relative longevity of the affluent [2, 20].

It is thus our view that a full answer to the question “What is aging” must, in the human case, recognize that aging is an unavoidably flexible and modifiable process that is continually reconstituted for individuals and for social groups and populations. While humans are quite unique in engaging in reflection upon and making efforts to change their own patterns of aging, most of the ongoing processes of continually reconstituting aging operate in terms that are taken for granted and uninterrogated, and that reproduce not only patterns of aging but the broader social arrangements that support those patterns. Again, consider the unintended impacts of social change and social conditions in the global growth in longevity and the demographic and epidemiological transitions. When deliberate human innovations are implemented, they are inevitably informed by the prevailing assumptions governing aging in the cultural settings in which they occur, and may institutionalize age-unfriendly aspects of those assumptions, whether in the ageism often built into the structure and culture of nursing homes, or the too-often, the unwarranted stigmatizing effects of age regimentation and age-based performance metrics in early childhood education.

These often implicit, culture-specific aging paradigms can be detected at a population or policy level, but remaining at the macro-structural domain may obscure the realities of “life on the ground”, where individuals encounter the stratified opportunities and constraints that ultimately produce a diverse range of outcomes within societies even while simultaneously producing new population-level trends.

Theories of human aging and many of the standard research paradigms they inform too infrequently consider either the social origins of observed patterns of aging in politically protected economic arrangements or  the power inherent in human activity itself, to reflect upon and potentially reshape, the habits, taken for granted patterns and social arrangements that organize the practices that produce observed patterns of aging, and the age-related expectations and rules that govern everyday life. An adequate understanding of human aging cannot avoid confronting such relentless and irreducible dynamics that produce the variation in such patterns.

## Data Availability

All data supporting the findings of this work are included in the article.

## Declarations

For this article no studies with human participants or animals were performed by any of the authors. All studies mentioned were in accordance with the ethical standards indicated in each case.

## References

[1] Achenbaum A (2015) A history of ageism since 1969. Generations 39(3):10–16

[2] Baars J (2023) Long lives are for the rich: aging, the life course, and social justice. Routledge, New York

[3] Baltes P (1979) Life-span developmental psychology: Some converging observations on history and theory. In Life-span Development and Behavior, Vol. 2, edited by Paul B. Baltes and Orville G. Brim, Jr., 255–279. New York: Academic Press.

[4] Baltes M, Wahl H‑W (1992) The dependency-support script in institutions: Generalization to community settings. Psychology and Aging 7(3):409–418 1388862 10.1037//0882-7974.7.3.409

[5] Bateson, MC (2001) Composing a life. New York: Grove Press.

[6] Bengston V (1973) The social psychology of aging. The Bobbs-Merrill Company, Indianapolis

[7] Berger P, Luckmann T (1967) The social construction of reality: A treatise in the sociology of knowledge. New York, NY: Penguin Books.

[8] Bernstein, Carl. 1969. “Age and race: fears seen in housing opposition.” Washington Post, (March 7th, 1969).

[9] Blumer H (1969) Symbolic interactionism: Perspective and method. Englewood Cliffs, NJ: Prentice-Hall.

[10] Bourdieu P (1977) Outline of a theory of practice. Translated by Richard Nice. Cambridge: Cambridge University Press.

[11] Bourdieu P (2017) Habitus. In J. Hillier & E. Rooksby (Eds.), Habitus: A sense of place (2nd ed., pp. 59–66). New York, NY: Routledge.

[12] Bourdieu P, Passeron J‑C (1970) Reproduction in education, society and culture. Translated by Richard Nice. London: Sage Publications.

[13] Boyd, C (2008) The Providence Mount St. Vincent experience. Journal of Social Work in Long Term Care 2(3-4): 245-268.

[14] Brent R (2022) Life expectancy in nursing homes. Applied Economics 54(16):1877–1888

[15] Broughton J (ed) (1987) Critical theories of psychological development. New York, NY: Plenum Press.

[16] Burawoy M (2019) Symbolic violence: conversations with Bourdieu. Duke University Press

[17] Butler R (1968) Ageism: another form of bigotry. The Gerontologist 9(4):243–246 10.1093/geront/9.4_part_1.243 5366225

[18] Butler R (1975) Why survive? Being old in America. New York, NY: Harper & Row.

[19] Cain L (1979) Adding Spice to Middle Age. Contemporary Sociology 8:547–550

[20] Case A and Deaton A (2020) Deaths of despair and the future of capitalism. Princeton,NJ: Princeton University Press.

[21] Castle N, Engberg J (2009) The health consequences of using physical restraints in nursing homes. Medical Care 47(11):1164–1173 19786918 10.1097/MLR.0b013e3181b58a69

[22] Castle N, McCaffrey D (2008) Staff turnover and quality of care in nursing homes. Medical Care 46(9):921–928 10.1097/01.mlr.0000163661.67170.b9 15908857

[23] Crystal, S, Shea DG and Reyes, AM. (2017) Cumulative advantage, cumulative disadvantage and evolving patterns of late life inequality. Gerontplogist 57(5):910-920 (Oct). 10.1093/geront/gnw056 PMC5881660 27030008

[24] Dannefer D (1984) Adult development and social theory: A paradigmatic reappraisal. American Sociological Review 49:100–116

[25] Dannefer D (2003) Cumulative advantage/disadvantage and the life course: Cross-fertilizing age and social science theory. The Journals of Gerontology Series B: Psychological Sciences and Social Sciences 58(6):S327–S337 14614120 10.1093/geronb/58.6.s327

[26] Dannefer D (2008) The waters we swim: Everyday social processes, micro-structural realities and human aging. Pp. 3-22 in Schaie KW and Abeles RP (eds), Social structures and aging individuals: Continuing challenges. New York: Springer.

[27] Dannefer D (2015) Right in front of us: taking everyday life seriously in the study of human development. Research in Human Development 12(3-4):209-216.

[28] Dannefer D (2021) Age and the reach of sociological imagination: Power, ideology, and the life course. New York: Routledge.

[29] Dannefer D, Perlmutter MA (1990) Human development as a multi-dimensional process: Individual and social constituents. Human Development 33(2–3):108–137

[30] Dannefer D, Stein P (1999) From the top to the bottom, from the bottom to the top: Systemically changing the culture of nursing homes. Final project report to the Van Ameringen Foundation

[31] Dannefer, D, Lin J and Gonos G (2021) Age-differentiated vs. age-integrated: Neoliberal policy and the future of the life course. Journal of Elder Policy 1(2):59-82.

[32] Deci E, Ryan R (2008) Self-determination theory: A macrotheory of human motivation, development, and health. Canadian psychology/Psychologie canadienne 49(3):182

[33] Diamond T (1992) Making gray gold: Narratives of nursing home care. University of Chicago Press, Chicago, IL

[34] Ekerdt D (1986) The busy ethic: Moral continuity between work and retirement. The Gerontologist 26(3):239–244 3721229 10.1093/geront/26.3.239

[35] Engberg J, Castle N, McCaffrey D (2008) Physical restraint initiation in nursing homes and subsequent resident health. The Gerontologist 48(4):442–452 18728294 10.1093/geront/48.4.442

[36] Falletta L, Dannefer D (2014) The life course and the social organization of age. In J Macleod, EJ Lawler and M Schwalbe (Eds). Handbook of the social psychology of inequality (pp. 607–625). Springer: Dordrecht, Netherlands.

[37] Ga H (2020) Long-term care system in Korea. 2020. Annals of geriatric medicine and research 24(3):181 32842717 10.4235/agmr.20.0036 PMC7533195

[38] George L, Ferraro K (2016) Aging and the social sciences: Progress and prospects. Handbook of aging and the social sciences, pp.3-22. Academic Press

[39] Gilligan C (1982) In a different voice: Psychological theory and women’s development. Cambridge, MA: Harvard University Press

[40] Goerres A, Vanhuysse P (2021) Global political demography: The politics of population change. Springer International Publishing, Cham, Switzerland

[41] Goffman E (1961) Asylums: Essays on the social situation of mental patients and other inmates. Garden City, NY: Anchor Books.

[42] Gubrium J (1976) Notes on the social organization of senility. Urban life 7(1):23–44

[43] Gullette MM (2011) Agewise: fighting the new ageism in America. University of Chicago Press, Chicago

[44] Hakimjavadi R, Yin C, Scott M et al (2025) Cognitive and functional decline among long-term care residents. JAMA Network Open 8(4):e255635 40266620 10.1001/jamanetworkopen.2025.5635 PMC12019527

[45] Harris GG (1978) Casting out anger: religion among the Taita of Kenya. Cambridge University Press, Cambridge

[46] Hochschild AR (1975) Disengagement theory: A critique and proposal. American Sociological Review 40(5):553–569

[47] Holstein JA and Gubrium JF (2000) Constructing the life course. Lanham,MD: Altamira Press.

[48] Joas H (1996) The creativity of action. Cambridge, UK: Polity Press.

[49] Katz S (2000) Busy bodies: Activity, aging, and the management of everyday life. Journal of Aging Studies 14(2)135–152.

[50] Kayser-Jones J (1990) Old, alone and neglected: Care of the aged in Scotland and the United States. Berkeley: University of California Press.

[51] Killen M and Smetana JG (2022) Handbook of moral development. New York: Routledge.

[52] Kushner E (2006) “Dr. Johnson on our Ageheimer’s”. https://www.ellen-kushner.livejournal.com/43698.html.

[53] Langer E, Rodin J (1976) The effects of choice and enhanced personal responsibility for the aged: A field experiment in an institutional setting. Journal of Personality and Social Psychology 34(2):191–198 10.1037//0022-3514.34.2.191 1011073

[54] Lerner R (2021) Individuals as producers of their own development: The dynamics of person–context coactions. New York: Routledge.

[55] Levinson DJ and Levinson JA (1996) Seasons of a woman’s life. New York: Alfred A. Knopf.

[56] McNees, Patrick (1997). A day in the life of a nursing home resident, or the lack thereof. Paper presented at the founding conference of the Pioneer Network. Rochester, NY.

[57] Irving Medical Center, Columbia University (2022) “Robert N. Butler: pioneer in study of aging”. https://www.cuimc.columbia.edu/news/robert-n-butler-pioneer-study-aging

[58] Montagu A (1989) Growing young. Bergin & Garvey, South Hadley, MA

[59] Morss J (1991) The biologising of childhood: Developmental psychology and the Darwinian myth. Routledge.

[60] Piaget, J (1970) Genetic epistemology. New York: Columbia.

[61] Rogoff B (2003) The cultural nature of human development. Oxford University Press, New York

[62] Rosenbaum J (1983) Career mobility in a corporate hierarchy. New York: Academic.

[63] Sapolsky R (2011) Zeitgeist: Moving Forward. https://www.youtube.com/watch?v=4Z9WVZddH9w

[64] Schaie W (1965) A general model for the study of developmental problems. Psychological bulletin 64(2):92–92 14320080 10.1037/h0022371

[65] Schmidt S (2020) Midlife crisis: the feminist origins of a chauvinist cliché. University of Chicago Press, Chicago

[66] Shura R, Siders R, Dannefer D (2011) Culture change in long-term care: Participatory action research and the role of the resident. The Gerontologist 51(2):212–225 21163911 10.1093/geront/gnq099 PMC3140257

[67] Skinner R, Yu J, Unruh M, Braun RT, Jung H‑Y, Johnson P, Fernandez R, Stevenson D (2025) State variation in staffing and characteristics of nursing homes most impacted by new federal standards. Health Affairs Scholar 3(8):qxaf154 40823006 10.1093/haschl/qxaf154 PMC12352297

[68] Stein, Paul. 2004. Social life under the evacuation of culture: lost minds, demented delves, and social solidarity. PhD dissertation, Warner Graduate School of Education and Huiman Development,, University of Rochester, Rochester, NY.

[69] Stetsenko A (2017) The transformative mind: Expanding Vygotsky’s approach to development and education. Cambridge University Press, Cambridge, UK

[70] Thomas W (1996) Life worth living: How someone you love can still enjoy life in a nursing home: The Eden Alternative in action. VanderWyk&Burnham

[71] Vianna E, Stetsenko A (2019) Turning resistance into passion for knowledge with the tools of agency: Teaching-learning about theories of evolution for social justice among foster youth. Perspectiva 38(4):864–886

[72] Vygotsky, L Mind in society: (1978) The development of higher psychological processes. Cambridge, MA: Harvard

[73] Ziegenhain P (2021) Getting old before getting rich (and not fully realizing it): Premature ageing and the demographic momentum in Southeast Asia. In: Goerres A, Van Huysse P (eds) Global political demography: The politics of population change. Springer International Publishing, Cham, Switzerland

